# Crescent IgA Nephropathy and its association with anti-neutrophil cytoplasm antibody: what do we know?

**DOI:** 10.1590/2175-8239-JBN-2022-E001

**Published:** 2022-02-23

**Authors:** Welder Zamoner, Pâmela Falbo dos Reis, Vanessa dos Santos Silva

**Affiliations:** 1 Universidade Estadual Paulista “Júlio de Mesquita Filho”, Faculdade de Medicina de Botucatu, Botucatu, SP, Brasil.

IgA nephropathy (IgAN) is the leading cause of primary glomerulopathy in the world, and its presentation varies substantially, including an asymptomatic finding of hematuria and/or proteinuria, recurrent macroscopic hematuria or, more rarely, nephritic syndrome, nephrotic syndrome, or even rapidly progressive glomerulopathy (RPGN)^1^. Renal biopsy with the predominant finding of IgA on immunofluorescence microscopy is essential for its diagnosis. RPGN patients may have crescents, but they usually do not affect more than 50% of the glomeruli and are associated with a worse prognosis if left untreated. Crescents are more frequent in Henoch-Schönlein Purpura than IgAN. About 25% to 30% of patients with IgAN progress to chronic kidney disease until its final stage and require renal replacement therapy^1^. Proteinuria above 1.0g/24h, elevated serum creatinine, arterial hypertension, and the MEST-C score are prognostic factors in IgAN. They have been organized into an internationally recognized tool to help physicians and patients calculate the prognosis and decide on treatment^2^. However, crescents were not added to this tool, and it is still a factor that does not fit in the most common manifestations in IgAN.

We have understood more clearly the pathogenesis of IgAN over the last few decades. There is already a consensus:it is a glomerulopathy caused by immune complex deposition. IgA1 deficient in galactose forms immune complexes with other IgAs, IgGs, soluble CD89, or food antigens^3^. These immune complexes are preferentially deposited in the mesangium after binding to particular receptors, activating mesangial cells and the complement system locally, leading to mesangial proliferation. Crescents are rare findings, and denote greater aggression to the endothelium of the glomerular capillary loop.

Pauci-immune small vessel vasculitis with positive anti-neutrophil cytoplasm antibody (ANCA) usually presents with renal involvement concomitant with lesions in the upper and/or lower airways and, more rarely, neurological damage and skin and eye damage. In these cases, there is no significant presence of immunoglobulins in the glomeruli upon immunofluorescence microscopy and fibrinoid necrosis in glomerular capillary loops is commonly seen, and the pathogenic role of ANCA is already recognized. It is known that a genetic predisposition linked to HLA class II which, added to environmental factors such as S. aureus infections, drug use (propylthiouracil, hydralazine, cocaine) and exposure to silica can trigger the formation of ANCA and initiate the glomerular and systemic aggression process^4^.

The association between IgAN and related ANCA-associated vasculitis is uncertain, with prevalence ranging from 1.2 to 5.8%^5,6^. Little is known about the clinical and prognostic importance of this combination. The presence of ANCA could amplify the inflammatory reaction^5^, with a worse prognosis being described for patients with this association, if not treated with immunosuppression^6,7^.

The study by Dias et al. (2021)^8^, presented for the first time a Brazilian series of patients with IgAN and the presence of a positive ANCA marker, arousing interest in understanding this association between IgA and ANCA. This study showed clinical and histological characteristics on presentation, pathophysiology, pathogenesis, and renal prognosis, by analyzing data from evolution of these patients throughout follow-up. Most of patients in the study had a percentage of crescents and renal disfunction since diagnosis, highlighting the greater severity of patients when IgAN is associated with the presence of ANCA. It remains unclear whether the association of IgAN and ANCA positivity is a coincidence or whether it is a new entity, and studies such as this one by Dias et al. (2021)^8^ can help to clarify points that are still unclear.

Another point of discussion is the treatment regimen, and some questions still instigate nephrologists: which immunosuppressive regimen should patients be treated? When would it be indicated? Previous studies show a better response to treatment when there are crescents in IgA and ANCA compared to IgA nephropathies with negative ANCA and no crescents^9,10^. In most studies, the patients had some systemic sign of vasculitis, and it is noteworthy that the patients in the study by Dias et al. (2021)^8^ did not show signs of involvement of the upper or lower airways, neurological or gastrointestinal tract.

In addition to contributing to a better understanding of this association between IgAN and related ANCA-associated vasculitis in our environment, the study awakens us to the need to systematically search for the presence of ANCA in patients with IgA^9^.
